# Novel Solution‐Processed Fe_2_O_3_/WS_2_ Hybrid Nanocomposite Dynamic Memristor for Advanced Power Efficiency in Neuromorphic Computing

**DOI:** 10.1002/advs.202408133

**Published:** 2025-03-09

**Authors:** Faisal Ghafoor, Honggyun Kim, Bilal Ghafoor, Zaheer Ahmed, Muhammad Farooq Khan, Muhammad Rabeel, Muhammad Faheem Maqsood, Sobia Nasir, Wajid Zulfiqar, Ghulam Dastageer, Myoung‐Jae Lee, Deok‐kee Kim

**Affiliations:** ^1^ Department of Electrical Engineering and Convergence Engineering for Intelligent Drone Sejong University Seoul 05006 Republic of Korea; ^2^ Department of Semiconductor Systems Engineering Sejong University Seoul 05006 Republic of Korea; ^3^ School of Materials Science and Engineering Shanghai University Shanghai 200444 China; ^4^ Material Science and Engineering Program College of Arts and Science American University of Sharjah Sharjah 26666 UAE; ^5^ Department of Physics and Astronomy Sejong University Seoul 05006 South Korea; ^6^ Institute of Conversion Daegu Gyeongbuk Institute of Science and Technology (DGIST) Daegu 42988 South Korea

**Keywords:** hybrid nanocomposite (HN), neuromorphic computing (NC), non‐volatile memory (NVM), transition‐metal dichalcogenides (TMDCs)

## Abstract

Non‐volatile memory (NVM) based neuromorphic computing, which is inspired by the human brain, is a compelling paradigm in regard to building energy‐efficient computing hardware that is tailored for artificial intelligence. However, the current state of the art NVMs are facing challenges with low operating voltages, energy efficiencies, and high densities in order to meet the new computing system beyond Moore's law. It is therefore necessary to develop novel hybrid materials with controlled compositional dynamics is crucial for initiating memristor devices capable of low‐power operations. This study validates the effectiveness of Ag/Fe_90_W_10_/Pt hybrid nanocomposite memristor devices, demonstrating superior performance including ultra‐low voltage operation, high stability, reproducibility, exceptional endurance (10^5^ cycles), environmental resilience, and low energy consumption of 0.072 pJ. Moreover, the memristor exhibits the ability to emulate essential biological synaptic mechanisms. The resistive switching phenomenon is primarily attributed to the controlled filament formation along unique heterophase grain boundaries. Furthermore, the hybrid nanocomposite synaptic device achieved an image recognition accuracy of 94.3% in Artificial Neural Network (ANN) simulations by using the Modified National Institute of Standards and Technology (MNIST) dataset. These results imply that the device's performance has promising implications for facilitating efficient neuromorphic architectures in the future.

## Graphical abstract

Solution‐processed approach for integration of Fe_2_O_3_/WS_2_ nano‐hybrid composite memristor devices. Remarkable switching characteristics and excellent durability for up to 10^5^ cycles. The device shows ultra‐low energy consumption of 0.072 pJ and excellent environmental stability. Memristor devices demonstrated high identification accuracy up to 94.3%.

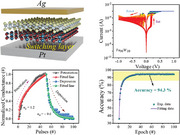

## 1 Introduction

The memristor represents a significant advance in the field of passive electrical components, offering significant advantages in integration density, operational speed, and power efficiency when compared to traditional resistors, capacitors, and inductors. The capacity to integrate memory and computation renders it a highly promising candidate for applications requiring the intelligence associated with the human brain.^[^ ^1^, ^2^, ^3^ ^]^ The memristor is typically configured with two electrode layers that are separated by an intermediate dielectric layer in a metal‐insulator‐metal structure.^[^ ^4^, ^5^ ^]^ The memristor can be categorized into two types, based on the continuity of resistance across the voltage sweep that is analog and digital memristors.^[^ ^6^ ^]^ The integration enables reconfigurable computing architectures to transition seamlessly between digital and analog processing, thereby increasing energy efficiency and speed. These advancements broaden the applicability of memristor‐based computing, enabling its integration into high‐precision neural networks and complementary metal–oxide–semiconductor (CMOS) compatible systems.^[^ ^7^, ^8^, ^9^ ^]^

Layered transition‐metal dichalcogenides (TMDCs), such as WS_2_, PdSe_2_, HfSe_2_, MoS_2_, and WSe_2_ offer tunable bandgaps, high carrier mobility, and precise thickness control due to their weak van der Waals bonding. However, achieving analog multilevel memory modulation for synaptic functions remains challenging due to controlled redox reactions near conductive filaments.^[^ ^10^, ^11^, ^12^, ^13^ ^]^ Among them tungsten sulfides (WS_2_) excel in memristor applications due to their tunable bandgap (1.2–2.1 eV) and superior electronic properties, including high electron mobility and low effective mass. Its exceptional interfacial characteristics enable the formation of stable van der Waals heterostructures, enhancing resistive switching through band structure modulation. WS_2_‐based devices exhibit controlled resistive switching via mechanisms like oxygen ion migration and trap‐controlled space charge limited current, facilitating the fabrication of high‐performance, low‐power memristive device.^[^ ^14^, ^15^, ^16^ ^]^ On the contrary, many oxides‐based memristor devices HfOx, TiO_2_, and WOx with different resistive switching characteristics have been studied.^[^ ^17^, ^18^, ^19^ ^]^ Iron (Fe) stands also out as a cost‐effective material due to its abundance as the fourth most abundant element in the earth's crust. Moreover, the characteristics of iron oxide (Fe_2_O_3_) make it a promising candidate for memristor devices, which offer potential advantages in terms of stability, compatibility, and scalability.^[^ ^20^, ^21^ ^]^ Fe_2_O_3_‐based ReRAM devices show promising synaptic properties but require further optimization of their resistive switching behavior. Enhancing endurance and retention properties is crucial to improving their practical applicability and unlocking their full potential in‐memory technologies.^[^ ^22^ ^]^ The recent studies propose solutions, such as the bilayer structure of ZrO_2_@ MoS_2_^[^ ^23^ ^]^ by introducing an interfacial TiOx layer on defective Mo‐irradiated ReS_2_^[^ ^24^ ^]^ and employing O_2_‐plasma treatment on HfSe_x_O_y_/HfSe_2_ for a controlled resistive switching filament formation.^[^ ^25^ ^]^ Oxygen plasma‐treated WSe_2−x_Oy with a graphene electrode and controlled oxidation of WSe_2_ in order to form a WO_3_–WSe_2_ interface also shows promise in regards to emulating synaptic functions.^[^ ^26^, ^27^ ^]^ These advancements hold potential for advancing neuromorphic systems via heterostructure‐based memristors and controlled oxide formation techniques. It is therefore crucial to develop efficient strategies in regard to regulating filament formation for the practical implementation of memristor devices based on 2D‐based hybrid nanocomposites. Solution‐processed nanocomposites via simplified sol–gel methods offer a cost‐effective, straightforward synthesis approach, particularly advantageous at lower temperatures.^[^ ^28^ ^]^

Neuromorphic computing systems represent a ground breaking approach in regard to constructing next‐generation computer architectures by offering parallel processing, high efficiency, and low power consumption.^[^ ^4^, ^29^ ^]^ The integration of robust analog memristors in these systems is crucial for building high‐performance neural networks, whereas the inclusion of digital memristors is also necessary to facilitate on‐chip logic operations. The recent efforts focused on attaining a digital‐to‐analog transition within single memristive systems,^[^ ^30^, ^31^, ^32^ ^]^ which is usually uncommon. The present study focused to develop highly uniform switching characteristics of memristors having low energy consumption, high stability, and forming free properties.

Fe_(1‐x)_W_(x)_ (x = 0, 10, 20 wt%) hybrid nanocomposites were synthesized using a simplified sol–gel method, enabling precise control over compositional dynamics. Memristors with different compositions of switching layers were fabricated, which included Ag/Fe_2_O_3_/Pt, Ag/Fe_90_W_10_/Pt, and Ag/Fe_80_W_20_/Pt. The Ag/Fe_90_W_10_/Pt memristor device showed extraordinary electrical performance and synaptic functionality among other devices. The device exhibited fast switching speed, high durability, expectational retention capabilities, low switching voltage, and synaptic characteristics. Furthermore, the device also exhibits multilevel storage characteristics with precise control over the reset voltage. Additionally, Short‐term plasticity (STP) and long‐term plasticity (LTP) features are successfully mimicked by the device capacity. The device's architecture also facilitates multipattern memorization, highlighting its ability to emulate the fundamental characteristics of biological synapses, which makes it possible to integrate sophisticated features in neuromorphic computing systems.

## 2 Experimental

WS_2_ and Fe_2_O_3_ nanoparticles were used for the synthesis of the Fe_2_O_3_/WS_2_ hybrid nanocomposites. For each composition, appropriate amounts of Fe_2_O_3_ and WS_2_ nanoparticles were measured by using a weight balance. The stoichiometric amounts of nanoparticles were added to the desired quantity of ethylene glycol and placed on a hot plate for stirring. Following thorough dispersion of nanoparticles in the solvent, the reaction was initiated by elevating the temperature to 80 °C. The temperature was subsequently raised to 120 °C, which led to the formation of a gel after two to three hours of heating. Finally, the hybrid nanocomposite nanoparticles were formed by raising the temperature to 200 °C on a hot plate. The labeled Fe_90_W_10_ sample is composed of 90% by weight of Fe_2_O_3_ nanoparticles and 10% by weight of WS_2_ nanoparticles.

The device structure comprised platinum (Pt) substrates as a bottom electrode that underwent a cleaning process with distilled water rinse and subsequent ultrasonication in acetone. The washed substrates were then dried at 100 °C in an oven prior for further processing. The switching layer of 40 nm thickness of Fe_2_O_3_/WS_2_ nanocomposite was deposited onto the substrates via thermal evaporation. The deposited films were annealed at 750 °C in a box furnace for two hours. Finally, the top electrode of 150 nm thick silver (Ag) was deposited by thermal evaporator. The synthesis and device fabrication process are shown in Figure S1 (Supporting Information).

## 3 Results and Discussion

**Figure 1:**
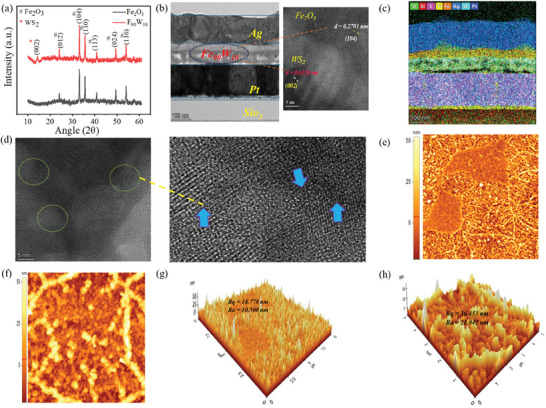
a) X‐ray diffraction examination of pure Fe_2_O_3_ and Fe_90_W_10_ hybrid nanocomposite samples. b) Cross‐sectional transmission electron microscopy (TEM) image of the Ag/Fe_90_W_10_/Pt structural device, (where the oval box matches to the Fe_2_O_3_ and WS_2_ lattice spacing c) scanning transmission electron microscopy (STEM) mapping for the elemental analysis of the Fe_90_W_10_ hybrid nanocomposite. d) STEM‐ADF image top‐view atomic resolution (The Fe_90_W_10_ FFT image was obtained using GMS3 software. The zoomed‐in image displays the lattice disordered area, which is marked by blue arrows). e,f) 2D AFM images of Fe_2_O_3_ and Fe_90_W_10_ hybrid nanocomposite. g,h) The 3D AFM images of the pure and Fe_90_W_10_ hybrid nanocomposites.

X‐ray diffraction analysis was performed to assess the crystalline structure and phase homogeneity of the synthesized nanocomposite, with diffraction patterns measured over a 2θ range of 10–60° as shown in **Figure** **1**a. The results revealed that the characteristic peaks that correspond to the Fe_2_O_3_ nanoparticles with the highest intensity peak were examined at 2θ ≈ 33.1, which matches the (104) plane, that is indicative of the hematite phase (JCPDS No.00‐024‐0072). The peaks that correspond to WS_2_ were also observed at 14.01, which is consistent with the hexagonal structure (JCPDS No. 87–2417).^[^ ^33^ ^]^ The presence of both WS_2_ and Fe_2_O_3_ phases was clearly observed in the Fe_2_O_3_‐WS_2_ hybrid nanocomposites. The Raman spectra of Fe_2_O_3_ nanoparticles and Fe_90_W_10_ nanocomposite, were measured in the range of 200–900 cm^−1^. The spectral analysis provides crucial vibrational information, enabling the characterization and comparison of the structural properties of these nanomaterials as shown in Figure S2 (Supporting Information). Transmission electron microscopy (TEM) was utilized for a detailed investigation of the Fe_90_W_10_ hybrid nanocomposites in order to elucidate their uniform resistive switching (RS) behavior. The cross‐sectional highresolution transmission electron microscopy (HRTEM) image in Figure 1b clearly illustrates the layered structure of the memristor device. By using Gaetan Software lattice spacings 0.270 and 0.612 nm of Fe_2_O_3_ and WS_2_ was determined that correspond to the (104) and (002) crystal plane of Fe_90_W_10_ hybrid nanocomposite. The presence of both nanoparticles was confirmed by these lattice spacings, and no additional phases or orientations were observed in the active layer. Further validation of the essential components of the electronic synapse is provided by the scanning transmission electron microscopy (STEM) mapping, which is depicted in Figure 1c. The blue arrows in Figure 1d highlight the lattice disorder in the FFT image, which clearly shows dispersed lattice fringes that reflect strong interaction of Fe_2_O_3_/WS_2_ at the interface.^[^ ^34^ ^]^

The surface topography and roughness of pure Fe_2_O_3_ and Fe_90_W_10_ hybrid nanocomposites are visualized through Atomic Force Microscopy (AFM) images shown in Figure 1e,f respectively. Adsorption occurs at the grain boundaries of the samples after a week of exposure to air. The pure Fe_2_O_3_ nanoparticles lack well‐defined grain boundaries, whereas the WS_2_‐Fe_2_O_3_ hybrid nanocomposites display well‐defined grain boundaries. This is due to the presence of WS_2_, which contains numerous defects such as sulfur and tungsten vacancies, which result in well‐defined grain boundaries. The pollutants tend to preferentially adhere to these grain boundaries upon exposure to air, which renders them visible in the Atomic Force Microscopy (AFM) image. Figure 1g,h provides a 3D AFM image of the pure Fe_2_O_3_ and Fe_90_W_10_ hybrid nanocomposite. The analysis of the surface roughness shows that the pure Fe_2_O_3_ sample has a mean roughness (Ra) of 10.500 nm and a root mean square roughness (Rq) of 18 .778 nm. The Fe_90_W_10_ nanocomposite sample in contrast exhibits a higher mean roughness (Ra) of 28.942 nm and root mean square roughness (Rq) of 36.453 nm. The device's improved performance results are due to the existence of high‐density grain boundaries in the polycrystalline nanocomposites with nanoscale grain sizes.

**Figure 2:**
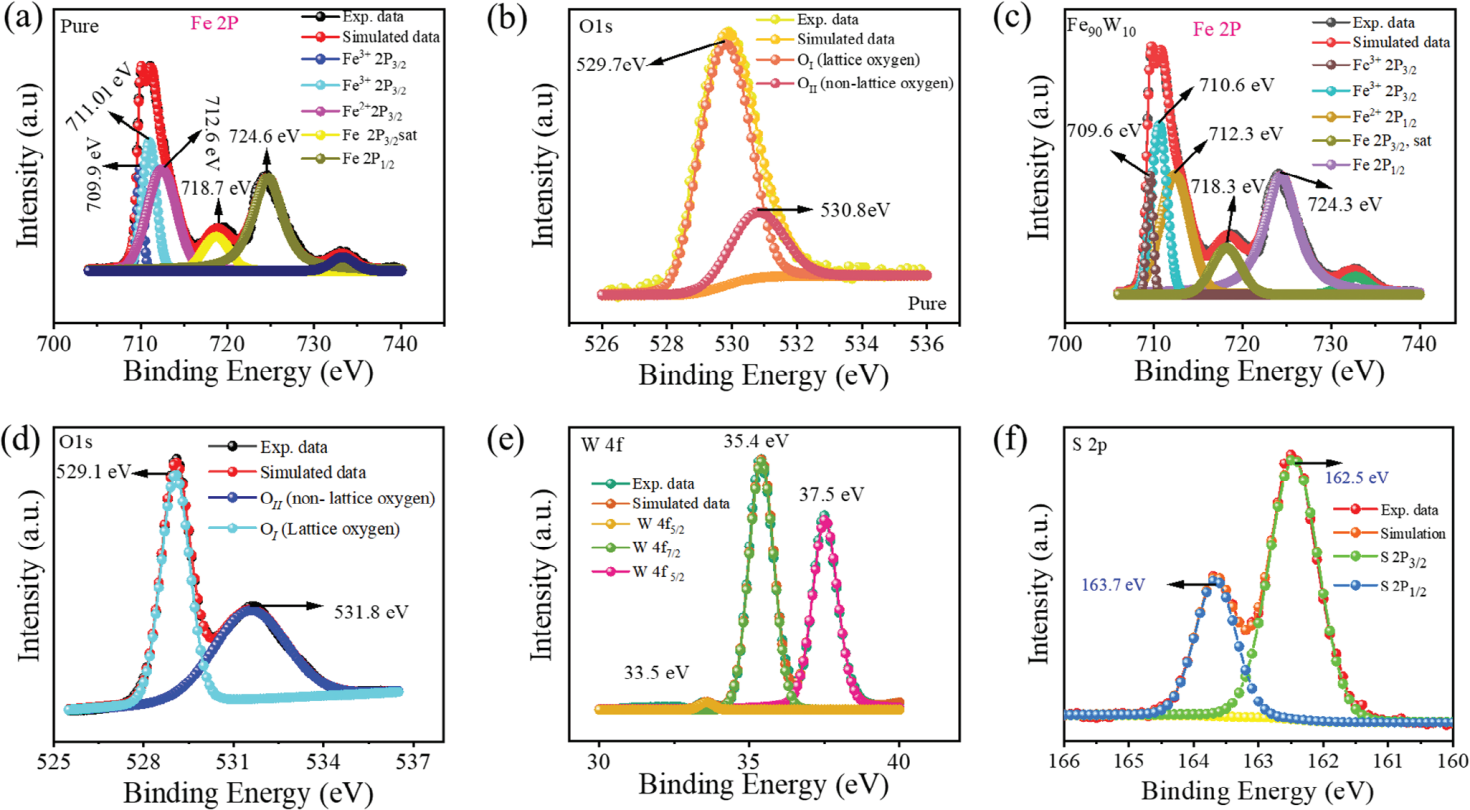
The Fe_2_O_3_ XPS analysis of a) Fe 2p and b) O1s, whereas the Fe_90_W_10_ hybrid nanocomposite's XPS spectra of c) Fe 2p, d) O 1s, e) W 4f, and f) S 2p.

X‐ray photoelectron spectroscopy (XPS) analysis, as shown in **Figure** **2**, was employed to elucidate the chemical states and composition of both the hybrid Fe_2_O_3_‐WS_2_ nanocomposite and pure Fe_2_O_3_. The nanoparticles of WS_2_ (100–400 eV) and Fe_2_O_3_ (700–900 eV) were identified as distinctive peaks in the hybrid nanocomposite spectra, which indicates the existence of both components in the nanocomposite. The Fe 2p peak and its satellite peaks of Fe 2p_1/2_ and Fe 2p_3/2_ provide valuable information regarding the ionic states of Fe.^[^ ^35^ ^]^ The Fe 2p peak was subjected to a deconvolution analysis in order to identify the Fe^2+^ or Fe^3+^ ions. The deconvoluted XPS Fe 2p, Fe 2p_3/2_, and Fe 2p_1/2_ XPS peaks for both the Fe_2_O_3_ and Fe_90_W_10_ hybrid samples are shown in Figure 2a,c. It is worth noting that in the spin–orbit (j–j) coupling, which is the Fe 2p_3/2_ peak that exhibits a fourfold degeneracy of the states, resulted in a smaller but stronger peak compared to the Fe 2p_1/2_ peak, which has a larger area and contains two states. Various studies examined the Fe 2p_3/2_ peak location, which yielded values that range from 710.6 to 711.2 eV.^[^ ^36^ ^]^ The oxygen peaks are observed in XPS spectrum of Fe_2_O_3_ at 530.8 and 529.7 eV, respectively corresponding to lattice oxygen and non‐lattice oxygen as shown in Figure 2b. The oxygen peaks of the Fe_90_W_10_ nanocomposites are analyzed at 529.1 and 531.8 eV, respectively. They undergo a chemical shift by exhibiting a lower binding energy (BE), as non‐lattice oxygen occupation inherently creates lattice site vacancies as shown in Figure 2d. The hybrid nanocomposite exhibited prominent tungsten (W) peaks at 30.1, 32.2, 34.6, and 36.6 eV, which indicates the presence of W in 4^+^,5^+^, and 6^+^ oxidation states that are shown in Figure 2e. This observation may be attributed to the interaction between the WS_2_ and Fe_2_O_3_ ions in the hybrid nanocomposite. The prominent sulfur peaks S^2−^ 2p_3/2_ and 2p_1/2_ states, respectively, were observed at 162.5 and 163.7 eV that confirm the presence of sulfur in the nanocomposite that is shown in Figure 2f.

**Figure 3:**
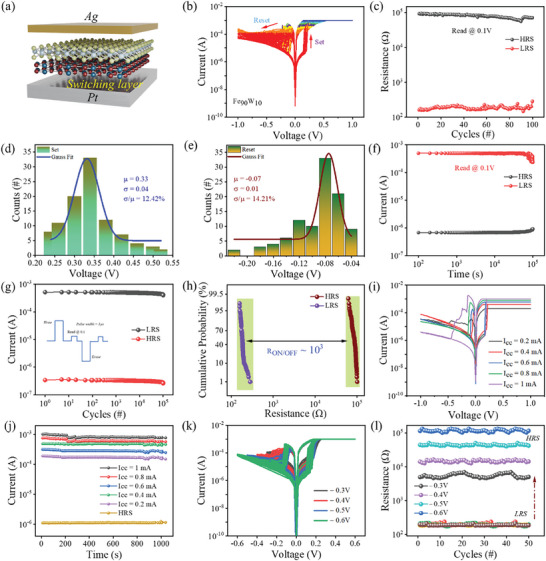
a) A Schematic illustration of the Fe_90_W_10_ memristor device. b) Multiple switching cycles of the Fe_90_W_10_ memristor device. c) Durability of F_90_W_10_ device after 100 switching cycles. d,e) The F_90_W_10_ hybrid nanocomposite's set and reset voltage histogram. f) Retention of the Fe_90_W_10_ hybrid nanocomposite up to 10^5^ s. g) Endurance of Fe_90_W_10_ up to 10^5^cycles. h) Cumulative probability of the switching cycles of the F_90_W_10_ hybrid nanocomposite. i) *I*–*V* curves of the Fe_90_W_10_ hybrid nanocomposite device at different compliance current that vary from 0.2, 0.4, 0.6, 0.8, and 1mA. j) Retention at different compliance currents of the device at a 0.1 read voltage for 10^3^ s. k) I–V curves with many levels at varying reset voltages (−0.30 to −0.60 V). l) Fe_90_W_10_ hybrid nanocomposite multi‐level endurance cycles at a 0.1 read voltage.

**Figure** **3**a depicts the Ag/Fe_90_W_10_/Pt device structure with platinum (Pt) as the bottom electrode, Fe_2_O_3_‐WS_2_ as a switching layer, and silver (Ag) as the top electrode (TE). The voltage is applied to the top electrode during the I–V measurements, whereas the bottom electrode, which is made from Pt, remains grounded. Current–voltage (I–V) characteristics were measured by applying bidirectional voltage sweeps in the sequence 0→−1V→0→1V→0. It was observed that the pure Fe_2_O_3_ memristors required significant electroforming compared to the hybrid nanocomposite memristors. The random generation of the Ag filaments resulted in a wide distribution of V set and V reset histograms in the Fe_2_O_3_ memristor device illustrated in Figure S3a–c (Supporting Information). On the other hand, the Ag/Fe_90_W_10_/Pt device showed consistent behavior with the switching cycles and the steady switching cycles without electroforming. The Ag/Fe_80_W_20_/Pt device also needed electroforming along with higher set/reset voltage and less stability, which is illustrated in Figure S4a,b (Supporting Information). The device exhibited bipolar resistive switching behavior, transitioning from a high resistance state (HRS) to a low resistance state (LRS) at 0.33 V during the positive sweep (set operation) and reverting to HRS at −0.07 V during the negative sweep (reset operation). The multiple switching cycles are shown in Figure 3b. The device's stability was assessed using endurance cycles conducted at a 0.1 read voltage, which are shown in Figure 3c. The device exhibited exceptional stability with minimal variations in both HRS and LRS. Figure 3d,e present statistical analyses of the set and reset voltages, including mean values (µ), standard deviations (σ), and coefficients of variation (Cv), demonstrating the consistency of the switching behavior. These values reveal a notable increase in the switching uniformity and a nearly ninefold decrease in the set and reset voltage fluctuation. The consecutive pulse endurance cycles and retention up to 1000 s are shown in Figure S5a,b (Supporting Information). Additionally, as Figure 3f,g illustrates, the memristor has exceptional retention qualities by being stable for more than 10^5^ s and enduring 10^5^ endurance cycles. The cycle‐to‐cycle variations were evaluated using a cumulative probability analysis, which is shown in Figure 3h. The device was also tested at various compliance currents that ranged from 0.2–1 mA, which are shown in Figure 3i. The results indicate superior and more controlled memristor characteristics at a 1 mA compliance current compared to lower current compliances. Retention analysis revealed that the low‐resistance state (LRS) experiences an increase in characteristic at different compliance currents, maintaining retention stability over 10^3^ s, whereas the high‐resistance state (HRS) remains relatively consistent as shown in Figure 3j. In addition, multiple stopping voltages were used to perform multi‐level I–V curve studies, and the device showed remarkable stability at all reset voltages for all cycles. The reset stopping voltage ranged from −0.30 to −0.60 V, which resulted in an increase in the high resistance state (HRS) current. The in situ endurance measures were also conducted to validate the device's multi‐level performance, which is shown in Figure 3k,l. The current–voltage (I–V) switching cycles of twelve fabricated devices were analyzed, demonstrating highly reproducible results as illustrated in Figure S6a–l (Supporting Information). Additionally, Figure S7a,b (Supporting Information) presents an evaluation of set and reset device‐to‐device variability across twelve fabricated memristive devices. Regarding the potential application of the Ag/Fe_90_W_10_/Pt memristor in neuromorphic computing, we conducted stability tests at reset stopping voltage ranging from −0.30 to −0.60 V on multiple devices as shown in Figure S8 (Supporting Information). To calculate the energy consumption per switch, we can use the given formula: W = V__pulse_ × I__pulse_ × t _pulse width_. Specifically, in this case, the set pulse has a current of 2.7 µA, a pulse width of 65 ns, and an amplitude of 0.5 V. The energy consumption per switch was 0.072 pJ, which is remarkable to use as a synaptic device as shown in Figure S9 (Supporting Information). These results suggest that the Ag/Fe_90_W_10_/Pt memristive device has superior stability and consistent switching behavior compared to Ag/Fe_2_O_3_/Pt memristive devices. The power consumption of the synaptic device compared with the other reported work is shown in Figure S10 (Supporting Information). Furthermore, the device has excellent environmental stability, and no degradation was observed after a six‐month measurement, which is shown in Figure S11 (Supporting Information). The Fe_90_W_10_ synaptic device exhibited remarkable advantages compared to the reported electronic synaptic devices, as shown in **Figure** **5a**.^[^ ^37^, ^38^, ^39^, ^40^, ^41^, ^42^, ^43^, ^44^, ^45^, ^46^, ^47^ ^]^ The Ag/Fe_90_W_10_/Pt memristive devices are therefore suitable for use as a multi‐level memory device and as a synergistic device for neuromorphic computing. The memristor conduction mechanism was consequently described by calculating the double logarithm of the HRS and LRS regions, which is illustrated in Figure S12 (Supporting Information). The resistive conduction mechanism of the devices may be understood by analyzing the ln(I) against ln (V) curves.^[^ ^48^ ^]^ The I–V characteristics show three unique slopes that correspond to three separate conduction mechanisms, which include ohmic conduction (slope = 1) and a space charge limited current mechanism (SCLC) with a limit of (slope = 2).

**Figure 4:**
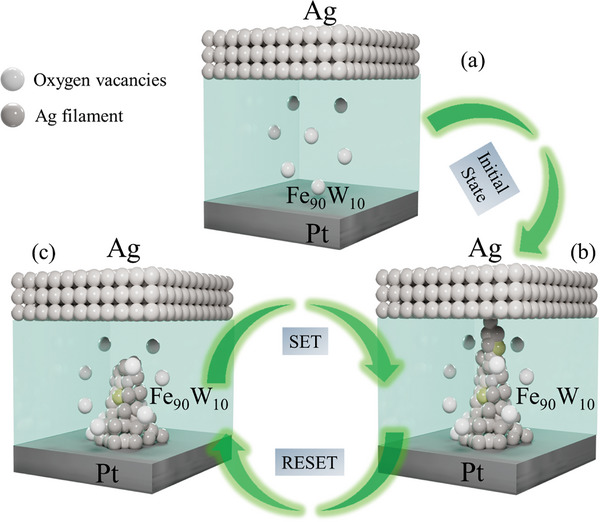
Switching process of F_90_W_10_ memristive device. a) Initial condition. b) Set process. c) Reset process.

The switching mechanism of the Ag/Fe_90_W_10_/Pt memristor is shown in **Figure** **4**. The switching mechanism in the Ag/Fe_90_W_10_/Pt ReRAM device can be elucidated by considering the device's structure and its electrical properties. The device initially remains in a stable state without any applied voltage, which is shown in Figure 4a.

The silver ions migrate toward the bottom electrode (Pt) under the influence of the electric field upon applying a positive voltage to the top electrode (Ag). Joule heating and thermophoresis effects consequently elevate the internal temperature, which leads to the formation of more vacancies in the S and W sites. The higher defect concentration in the low‐resistance state (LRS) facilitates easier electron hopping between vacancies, which thereby increases the overall conductance.^[^ ^49^, ^50^, ^51^ ^]^ Filament formation and conduction occur through a complex electrochemical process within the Fe_90_W_10_ hybrid nanocomposite switching layer. As a bias is applied, vacancies in both W and S positions of the WS_2_ layer act as dopants and facilitate ion migration. Ag+ ions from the top electrode move toward the bottom electrode, where they gain electrons and reduce to Ag atoms. These atoms accumulate to form prominent silver filaments, with their growth favored by the electric field distribution. The atomic‐scale silver filaments develop as Ag ions diffuse through the nanocomposite, resulting in characteristic electrochemical metallization (ECM) memristive behavior. As the voltage increases, the filaments extend further toward the Ag electrode, with some potentially reaching into the nanocomposite layer as a SET state shown in Figure 4b. Filament rupture, crucial for switching between resistance states, likely occurs when a reverse bias is applied, or the voltage drops below a certain threshold. The dissolution of filaments typically begins at their thinnest points, often near the active electrode interface as Reset state shown in Figure 4c. In this system, the grain boundaries of the Fe_90_W_10_nanocomposite play a vital role in constraining smaller Ag filaments, potentially serving as weak points where rupture is more probable. This interplay between filament formation, conduction, and rupture, mediated by the unique properties of the hybrid nanocomposite switching layer, enables the device's memristive functionality. The schematic representation of conducting and rapture of filament is also shown in Supplementary Figure S13 (Supporting Information).

**Figure 5:**
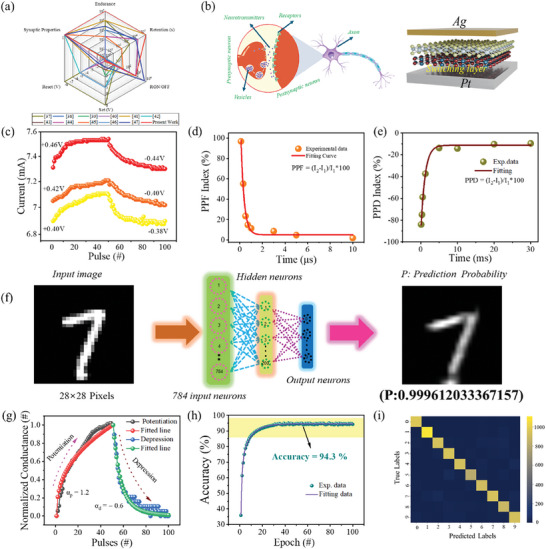
a) Performance metrics of the synaptic device compared with existing literature. b) A schematic depiction of synaptic elements in the human and artificial brains. (Left: pre‐ and post‐synaptic neurons functioning within a biological synapse; right: an artificial synapse employing Fe_90_W_10_ hybrid nanocomposite). c) By changing the pulse voltage from (0.40–0.46 V) during potentiation and (−0.38–0.44 V) during depression respectively the current change was observed. d,e) Paired‐pulse facilitation (PPF)% and paired‐pulse depression (PPD)% of Fe_90_W_10_ hybrid nanocomposite. f) An artificial neural network (ANN) flow chart was created for categorizing the MNIST dataset. g) The LTP and LTD characteristics are used to obtain the nonlinearity components (αp and αd) of normalized conductance. h) The accuracy of digit recognition was evaluated after 100 epochs. i) Handwritten MNIST training datasets digit classification results are shown in a confusion matrix.

Gradual switching plays a crucial role in regard to the advancement of neuromorphic synapses for neuromorphic applications. An increase of 0.1 V in the reset‐stop mechanism regulates the RESET procedure, which is depicted in Supplementary Figure S14 (Supporting Information). Figure 5b depicts the schematic structure of the Ag/Fe_90_W_10_/Pt memristor, to emulate biological synapses. The conductance modulation is observed under positive and negative pulse biases, which resemble the potentiation and depression of synaptic responses. This investigation focused on their ability to exhibit Long‐Term Potentiation (LTP) and Long‐Term Depression (LTD) behaviors under pulsed input signals with varying pulse amplitude to demonstrate the potential of Ag/Fe_90_W_10_/Pt memristors to mimicking synapses. The device was subjected to 50 potentiation pulses and 50 depression pulses, which are shown in Figure 5c. Both the potentiation and depression pulses had a width of 1 µs. A reading pulse of 0.1 V was used to measure the conductance changes after each set of pulses. The increase in resistance modulation occurs by increasing the pulse voltage (0.40–0.46) during the LTP process.^[^ ^52^ ^]^ The pulse voltages (−0.38 to −0.44) were carefully chosen to allow the resistance to return to its initial state to induce Long‐Term Depression (LTD). This approach aims to establish the appropriate conductance states within synapses and mimic linear weight‐updating behavior, which is critical for the development of neuromorphic networks.^[^ ^32^ ^]^ A potential indicator of the electronic synapse's performance effectiveness is the change in synaptic weight that was observed during LTD and LTP,^[^ ^53^ ^]^ which is shown in Figure 5g. The device nonlinearity (NL) can be calculated using Equation (1), which is provided below.

(1) $$\begin{array}{c} G={\left(\left(G_{\;\mathrm{LRS}}^{\alpha }-G_{\;\mathrm{HRS}}^{\alpha }\right)\times w+G_{\;\mathrm{HRS}}^{\alpha }\right)}^{1/\alpha }\;\mathrm{if}\;\alpha \neq 0\\ G=G_{\mathrm{HRS}}\times {\left(G_{\mathrm{LRS}}/G_{\mathrm{HRS}}\right)}^{w}\;\mathrm{if}\;\alpha =0 \end{array}$$

The conductance in the low‐resistance state (LRS) and the high‐resistance state (HRS) is represented by GLRS and GHRS. A vital nonlinearity aspect that critically controls either potentiation (αp) or depression (αd) features is the parameter α, which has a range of 0–1.^[^ ^54^ ^]^ The memristors exhibit superior linearity of the LTD and LTP because the conductive filament development is more linear at low voltages than it is at high voltages.^[^ ^55^ ^]^

Short‐term synaptic plasticity is a critical mechanism in neural networks, facilitating dynamic information processing and computational flexibility through transient modifications in synaptic efficacy. Short‐term synaptic plasticity manifests through Paired‐Pulse Facilitation (PPF) and Paired‐Pulse Depression (PPD), phenomena occurring when closely timed presynaptic spikes modulate subsequent synaptic responses. PPF is characterized by an enhanced excitatory postsynaptic current (EPSC) following a second spike, while PPD results in a diminished inhibitory postsynaptic current (IPSC). This study employed positive and negative paired presynaptic pulses to elicit EPSC and IPSC responses, respectively. The pulse scheme of PPF and PPD is shown in Supplementary Figure S15a,b (Supporting Information). The plasticity was quantified by analyzing the ratio of the second postsynaptic current (PSC) peak (I_2_) to the first PSC peak (I_1_), providing a measure of synaptic strength modulation. The response indices of PPF(%) and PPD (%)as a function of Δt, are shown in Figure 5d,e. The response index for PPF showed an exponential increase to 91% when Δt was short (Δt = 1 µs), whereas the exponential decreased to 41% for the PPF response. As the time interval (Δt) between pulses increased, both the PPF and PPD response indices gradually declined and eventually reached saturation levels, which mirrored biological synaptic behavior. The experimental paired‐pulse facilitation (PPF) index was fitted using a double exponential decay function, as described by the following equation:^[^ ^56^ ^]^

(2) $$\mathrm{PPF}\;\mathrm{index}\%=C_{1}\;\exp\left(\Delta t/\tau_{1}\right)+C_{2}\exp\left(\Delta t/\tau_{2}\right)$$

In this equation, C1 and C2 represent the initial facilitation constants for fast and slow decay processes of the excitatory postsynaptic current (EPSC), τ_1_ and τ_2_ denote the characteristic relaxation times, and Δt signifies the paired‐pulse delay time. From fitting the experimental data, the relaxation time constants were extracted as 0.16 and 3.13 µs, which are consistent with the behavior of biological synapses.

The application of various Long‐Term Potentiation (LTP) and Long‐Term Depression (LTD) processes on a neural network structure with dimensions 784 × 200 × 10 was conducted to evaluate the accuracy of the MNIST handwriting recognition dataset. The input neurons are standardized and transformed into a 1D array (784 × 1), which represents the 28 × 28 pixels. The neural network comprises 200 hidden layers that are interconnected with the input and output layers. Each of the ten output neurons corresponds to a distinct class of digits in the MNIST database. The nodes within the synaptic array of memristors align with individual neurons in the neural network. A schematic diagram that illustrates the neural network process is shown in Figure 5f. The recognition performance of an Artificial Neural Network (ANN) was evaluated using each class probability shown in the Figure S16 (Supporting Information). The prediction probability for class 7 is maximized, indicating the synaptic device's strong recognition capability. A recognition rate of 94.3% was attained with the MNIST test images, which are shown in Figure 5h, by following 100 epochs of ANN training. The additional simulations that account for device‐to‐device variability, including fluctuations and learning rate in parameters are shown in Figure S17 (Supporting Information). As the number of epochs increased, the conductance (G) became more uniform. The color change along the diagonal line in the confusion matrix, which is shown in Figure 5i, illustrates the incremental learning process of the neural network during pattern recognition. Each color corresponds to a specific value that indicates the inferred output. Successful recognition was achieved after 100 epochs, which is proven by the consistent alignment of the maximum inferred output value in each row with the desired output in each column. These findings highlight the significant potential of the proposed hybrid nanocomposite ANN for pattern recognition tasks.

## 4 Conclusion

In summary, we successfully fabricated an Ag/Fe_90_W_10_/Pt non‐volatile memristor by improving the switching speed, durability, and retention capability via control compositional dynamics. The device demonstrates remarkable stability over time, which includes a wide memory window of ≈10^3^, a low energy consumption of 0.072 pJ, fast switching speed, and a low Vset of ≈0.33 V, alongside ultra‐long data retention capabilities lasting ≈10^6^ s. This prolonged data retention can be attributed to the strong diffusion effect and robust interfacial contact at the interface. Moreover, the device successfully emulates the key biological synaptic activities, which include learning and memory processes, which were observed during paired‐pulse facilitation (PPF), paired‐pulse depression (PPD), long‐term potentiation (LTP), long‐term depression (LTD) transitions and MNIST recognition of ≈94.3%. This implies that the cost‐effective Ag/Fe_90_W_10_/Pt memristors have significant potential in regard to advancing brain‐inspired computing in the future.

## Conflict of Interest

The authors declare no conflict of interest.

## Author Contributions

F. G. and H. K. contributed equally to this work. F. G. contributed to writing the original draft, data curation, material synthesis, visualization, and formal analysis. H. K. performed methodology, investigation, and visualization. B. G. Performed methodology, investigation, and data curation. Z. A. performed methodology and Investigation, while Md. F. K. performed methodology and investigation. Md. R. participated in the review and Methodology, while Md. F.M. performed methodology and investigation. S. N. performed methodology and Investigation. W. Z. performed investigation and visualization. G. D. performed validation and visualization. M‐J. L. performed conceptualization, review, and visualization. D.‐K. K. performed conceptualization, supervision, and wrote–reviewed, and edited the final manuscript.

## Supporting information



Supporting Information

## Data Availability

The data that support the findings of this study are available on request from the corresponding author. The data are not publicly available due to privacy or ethical restrictions.

## References

[1] L. Chua , IEEE Trans. Circuit Theory 1971, 18, 507.

[2] J. Li , Z. Shen , Y. Cao , X. Tu , C. Zhao , Y. Liu , Z. Wen , Nano Energy 2022, 103, 107744.

[3] J. Gong , H. Wei , J. Liu , L. Sun , Z. Xu , H. Huang , W. Xu , Matter 2022, 5, 1578.

[4] Q. Liu , S. Gao , L. Xu , W. Yue , C. Zhang , H. Kan , Y. Li , G. Shen , Chem. Soc. Rev. 2022, 51, 3341. 35293907 10.1039/d1cs00886b

[5] J. Tang , F. Yuan , X. Shen , Z. Wang , M. Rao , Y. He , Y. Sun , X. Li , W. Zhang , Y. Li , Adv. Mater. 2019, 31, 1902761. 10.1002/adma.201902761 31550405

[6] Y. Wang , W. Wang , C. Zhang , H. Kan , W. Yue , J. Pang , S. Gao , Y. Li , ACS Appl. Electron. Mater. 2022, 4, 3525.

[7] K. Portner , M. Schmuck , P. Lehmann , C. Weilenmann , C. Haffner , P. Ma , J. Leuthold , M. Luisier , A. Emboras , ACS Nano 2021, 15, 14776. 34459580 10.1021/acsnano.1c04654

[8] K. Wang , Y. Jia , X. Yan , Nano Energy 2022, 100, 107486.

[9] M. Rao , H. Tang , J. Wu , W. Song , M. Zhang , W. Yin , Y. Zhuo , F. Kiani , B. Chen , X. Jiang , Nature 2023, 615, 823. 36991190 10.1038/s41586-023-05759-5

[10] H. Wang , D. Ren , C. Lu , X. Yan , Appl. Phys. Lett. 2018, 112, 231903.

[11] X. Wang , M. T. Pettes , Y. Wang , J.‐X. Zhu , R. Dhall , C. Song , A. C. Jones , J. Ciston , J. Yoo , Nano Lett. 2023, 23, 3754. 37094221 10.1021/acs.nanolett.2c04987

[12] S. Teja Nibhanupudi , A. Roy , D. Veksler , M. Coupin , K. C. Matthews , M. Disiena , Ansh, J. V. S. , I. R. Gearba‐Dolocan , J. Warner , Nat. Commun. 2024, 15, 2334. 38485722 10.1038/s41467-024-46372-y PMC10940724

[13] S. Parveen , L. T. Manamel , A. Mukherjee , S. Sagar , B. C. Das , Adv. Mater. Interfaces 2022, 9, 2200562.

[14] F. Huang , C. Ke , J. Li , L. Chen , J. Yin , X. Li , Z. Wu , C. Zhang , F. Xu , Y. Wu , Adv. Sci. 2023, 10, 2302813. 10.1002/advs.202302813 PMC10558669 37530215

[15] Q. Cao , P. Zou , P. Li , L. Xiong , H. Bi , J. Wu , J. Mater. Sci.: Mater. Electron. 2023, 34, 185.

[16] A. Thomas , P. Saha , M. Sahad E , N. Krishnan K , B. C. Das , ACS Appl. Mater. Interfaces 2024, 16, 20693. 10.1021/acsami.3c19177 38594622

[17] V. Milo , C. Zambelli , P. Olivo , E. Pérez , M. K. Mahadevaiah , O. G. Ossorio , C. Wenger , D. Ielmini , APL Mater. 2019, 7, 081120.

[18] D. P. Sahu , S. N. Jammalamadaka , Sci. Rep. 2019, 9, 16141. 31695093 10.1038/s41598-019-52522-w PMC6834672

[19] J. Go , Y. Kim , M. Kwak , J. Song , S. A. Chekol , J.‐D. Kwon , H. Hwang , Appl. Phys. Express 2019, 12, 026503.

[20] T. Ishibe , H. Matsui , K. Watanabe , S. Takeuchi , A. Sakai , Y. Nakamura , Appl. Phys. Express 2016, 9, 055508.

[21] T. Ishibe , T. Kurokawa , N. Naruse , Y. Nakamura , Appl. Phys. Lett. 2018, 113, 141601.

[22] Z. Sheykhifar , S. M. Mohseni , Sci. Rep. 2022, 12, 18771. 36335197 10.1038/s41598-022-23404-5 PMC9637095

[23] F. Wu , S. Si , P. Cao , W. Wei , X. Zhao , T. Shi , X. Zhang , J. Ma , R. Cao , L. Liao , Adv. Electron. Mater. 2019, 5, 1800747.

[24] M. E. Pam , S. Li , T. Su , Y. C. Chien , Y. Li , Y. S. Ang , K. W. Ang , Adv. Mater. 2022, 34, 2202722. 10.1002/adma.202202722 35610176

[25] L. Liu , Y. Li , X. Huang , J. Chen , Z. Yang , K. H. Xue , M. Xu , H. Chen , P. Zhou , X. Miao , Adv. Sci. 2021, 8, 2005038. 10.1002/advs.202005038 PMC8336485 34050639

[26] H.‐K. He , F.‐F. Yang , R. Yang , Phys. Chem. Chem. Phys. 2020, 22, 20658. 32895683 10.1039/d0cp03822a

[27] H.‐K. He , R. Yang , H.‐M. Huang , F.‐F. Yang , Y.‐Z. Wu , J. Shaibo , X. Guo , Nanoscale 2020, 12, 380. 31825449 10.1039/c9nr07941f

[28] C. J. Brinker , G. W. Scherer , J. Non‐Cryst. Solids 1985, 70, 301.

[29] N. Kumar , M. Patel , T. T. Nguyen , P. Bhatnagar , J. Kim , Mater. Today Chem. 2022, 23, 100681.

[30] H. J. Kim , Y.‐J. Baek , Y. J. Choi , C. J. Kang , H. H. Lee , H.‐M. Kim , K.‐B. Kim , T.‐S. Yoon , RSC Adv. 2013, 3, 20978.

[31] F. M. Simanjuntak , C.‐L. Hsu , T. Abbey , L.‐Y. Chang , S. Rajasekaran , T. Prodromakis , T.‐Y. Tseng , APL Mater. 2021, 9, 121103.

[32] A. Saleem , F. M. Simanjuntak , S. Chandrasekaran , S. Rajasekaran , T.‐Y. Tseng , T. Prodromakis , Appl. Phys. Lett. 2021, 118, 112103.

[33] S. P. Vattikuti , C. Byon , V. Chitturi , Superlattices Microstruct. 2016, 94, 39.

[34] S. Zuluaga , J. Lin , K. Suenaga , S. T. Pantelides , 2D Materials 2018, 5, 035025.

[35] S. Roosendaal , B. Van Asselen , J. Elsenaar , A. Vredenberg , F. Habraken , Surf. Sci. 1999, 442, 329.

[36] M. Muhler , R. Schlögl , G. Ertl , J. Catal. 1992, 138, 413.

[37] Z. Ren , G. Zhou , S. Wei , Phys. Chem. Chem. Phys. 2020, 22, 2743. 31984390 10.1039/c9cp06392g

[38] S. J. Shinde , M. R. Waikar , S. R. Gurav , S. L. Patil , S. D. Ghongade , A. M. Bagwan , A. R. Sonkawade , R. K. Sonker , R. K. Kamat , T. D. Dongale , Mater. Sci. Semicond. Process. 2024, 176, 108298.

[39] K. M. Lee , J. T. Jang , Y.‐J. Baek , H. Kang , S. Choi , S.‐J. Choi , D. M. Kim , C. J. Kang , T.‐S. Yoon , H.‐S. Mo , IEEE Electron Device Lett. 2016, 37, 986.

[40] P. Jetty , K. U. Mohanan , S. N. Jammalamadaka , Nanotechnology 2023, 34, 265703. 10.1088/1361-6528/acc811 36975196

[41] U. Das , S. Bhattacharjee , B. Mahato , M. Prajapat , P. Sarkar , A. Roy , Mater. Sci. Semicond. Process. 2020, 107, 104837.

[42] J.‐D. Kim , Y.‐J. Baek , Y. J. Choi , C. J. Kang , H. Ho Lee , H.‐M. Kim , K.‐B. Kim , T.‐S. Yoon , J. Appl. Phys. 2013, 114, 224505.

[43] H. H. Nguyen , H. K. T. Ta , S. Park , T. B. Phan , N. K. Pham , RSC Adv. 2020, 10, 12900. 35492079 10.1039/c9ra10101b PMC9051151

[44] J. H. Lee , C. Wu , S. Sung , H. An , T. W. Kim , Sci. Rep. 2019, 9, 19316. 31848387 10.1038/s41598-019-55637-2 PMC6917699

[45] N. He , Q. Zhang , L. Tao , X. Chen , Q. Qin , X. Liu , X. Lian , X. Wan , E. Hu , J. Xu , IEEE Electron Device Lett. 2021, 42, 319.

[46] S. Saha , V. Adepu , K. Gohel , P. Sahatiya , S. S. Dan , IEEE Trans. Electron Devices 2022, 69, 5921.

[47] M. Patel , D. D. Kumbhar , J. Gosai , M. R. Sekhar , A. T. Mallajosyula , A. Solanki , Adv. Electron. Mater. 2023, 9, 2200908.

[48] Y. C. Yang , F. Pan , Q. Liu , M. Liu , F. Zeng , Nano Lett. 2009, 9, 1636. 19271714 10.1021/nl900006g

[49] Q. Liu , S. Long , H. Lv , W. Wang , J. Niu , Z. Huo , J. Chen , M. Liu , ACS Nano 2010, 4, 6162. 20853865 10.1021/nn1017582

[50] L. Gao , Y. Li , Q. Li , Z. Song , F. Ma , Nanotechnology 2017, 28, 215201. 28462908 10.1088/1361-6528/aa6cd0

[51] Y.‐J. Huang , S.‐C. Chao , D.‐H. Lien , C.‐Y. Wen , J.‐H. He , S.‐C. Lee , Sci. Rep. 2016, 6, 23945. 27052322 10.1038/srep23945 PMC4823777

[52] X. Yan , L. Zhang , H. Chen , X. Li , J. Wang , Q. Liu , P. Zhou , Adv. Funct. Mater. 2018, 28, 1803728.

[53] P. Zhang , M. Xia , F. Zhuge , Y. Zhou , Z. Wang , B. Dong , Y. Fu , K. Yang , Y. Li , Y. He , Nano Lett. 2019, 19, 4279. 31150262 10.1021/acs.nanolett.9b00525

[54] J.‐W. Jang , S. Park , G. W. Burr , H. Hwang , Y.‐H. Jeong , IEEE Electron Device Lett. 2015, 36, 457.

[55] Y. Li , S. Chen , Z. Yu , S. Li , Y. Xiong , M. E. Pam , Y. W. Zhang , K. W. Ang , Adv. Mater. 2022, 34, 2201488. 10.1002/adma.202201488 35393702

[56] Y. N. Zhong , T. Wang , X. Gao , J. L. Xu , S. D. Wang , Adv. Funct. Mater. 2018, 28, 1800854.

