# Supplementary material for: Novel Solution‐Processed Fe2O3/WS2 Hybrid Nanocomposite Dynamic Memristor for Advanced Power Efficiency in Neuromorphic Computing
Source: Adv Sci (Weinh). 2025 Mar 9;12(17):2408133. doi: 10.1002/advs.202408133 (PMC12061252; doi:10.1002/advs.202408133)
Supplement: Supplementary file 1 — Supporting Information [file ADVS-12-2408133-s001.docx]

Novel Solution-Processed Fe_2_O_3_/WS_2_ Hybrid Nanocomposite Dynamic Memristor for Advanced Power Efficiency in Neuromorphic Computing.

Faisal Ghafoor^a,#^, Honggyun Kim^b,#^, Bilal Ghafoor^c^, Zaheer Ahmed^a,b^, Muhammad Farooq Khan^a^, Muhammad Rabeel^a^, Muhammad Faheem Maqsood^d^, Sobia Nasir^a,b^, Wajid Zulfiqar^a,b^, Ghulam Dastageer^e^, Myoung-Jae Lee^f*^, Deok-kee Kim^a,b^*

^a^ Department of Electrical Engineering and Convergence Engineering for Intelligent Drone, Sejong University, Seoul 05006, Republic of Korea

^b^ Department of Semiconductor Systems Engineering, Sejong University, Seoul 05006, Republic of Korea

^c^ School of Materials Science and Engineering, Shanghai University, Shanghai,200444, China.

^d^ Material Science and Engineering Program, College of Arts and Science, American University of Sharjah, Sharjah 26666, United Arab Emirates.

^e^ Department of Physics and Astronomy, Sejong University, Seoul 05006, Korea.

^f^ Institute of Conversion Daegu Gyeongbuk Institute of Science and Technology (DGIST), Daegu 42988, Korea.

#These authors contributed equally

*Corresponding E-mail:* *[deokkeekim@sejong.ac.kr](mailto:deokkeekim@sejong.ac.kr) & myoungjae.lee@dgist.ac.kr*


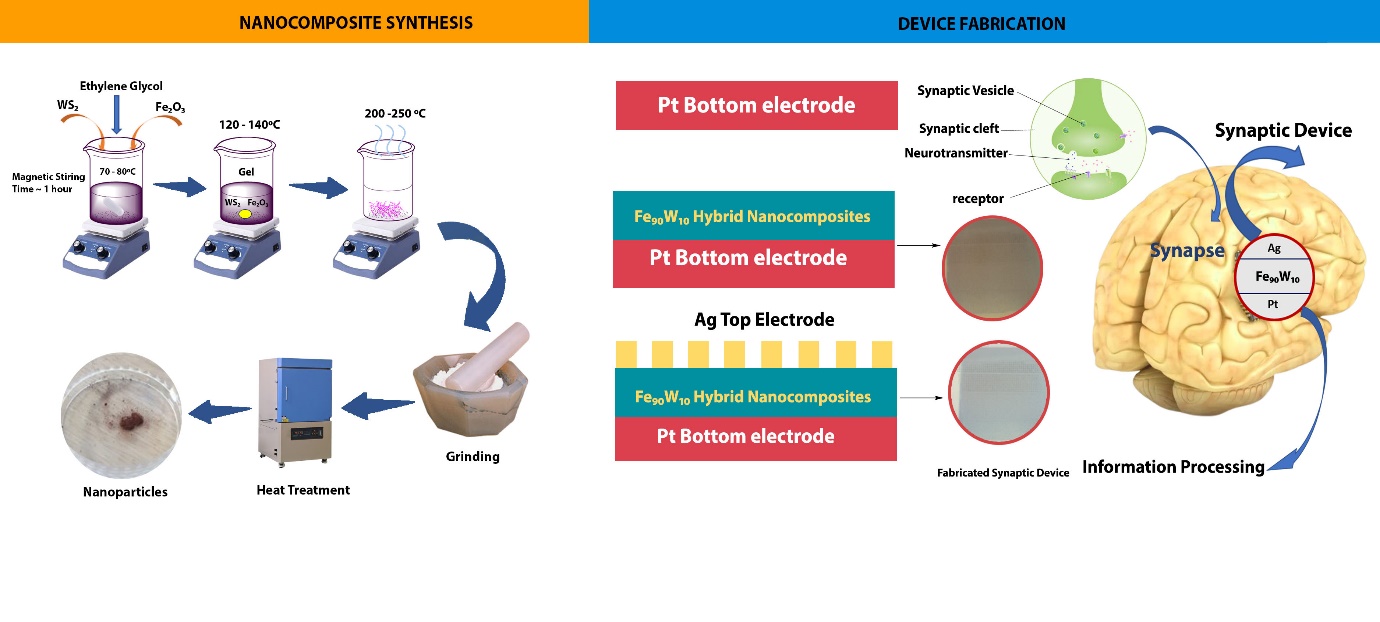


Figure S1: Synthesis and device fabrication of Ag/ Fe_90_W_10_/Pt hybrid nanocomposite.

Figure S2 : Raman analysis of pure Fe_2_O_3_ and Fe_90_W_10_ hybrid nanocomposite.

**
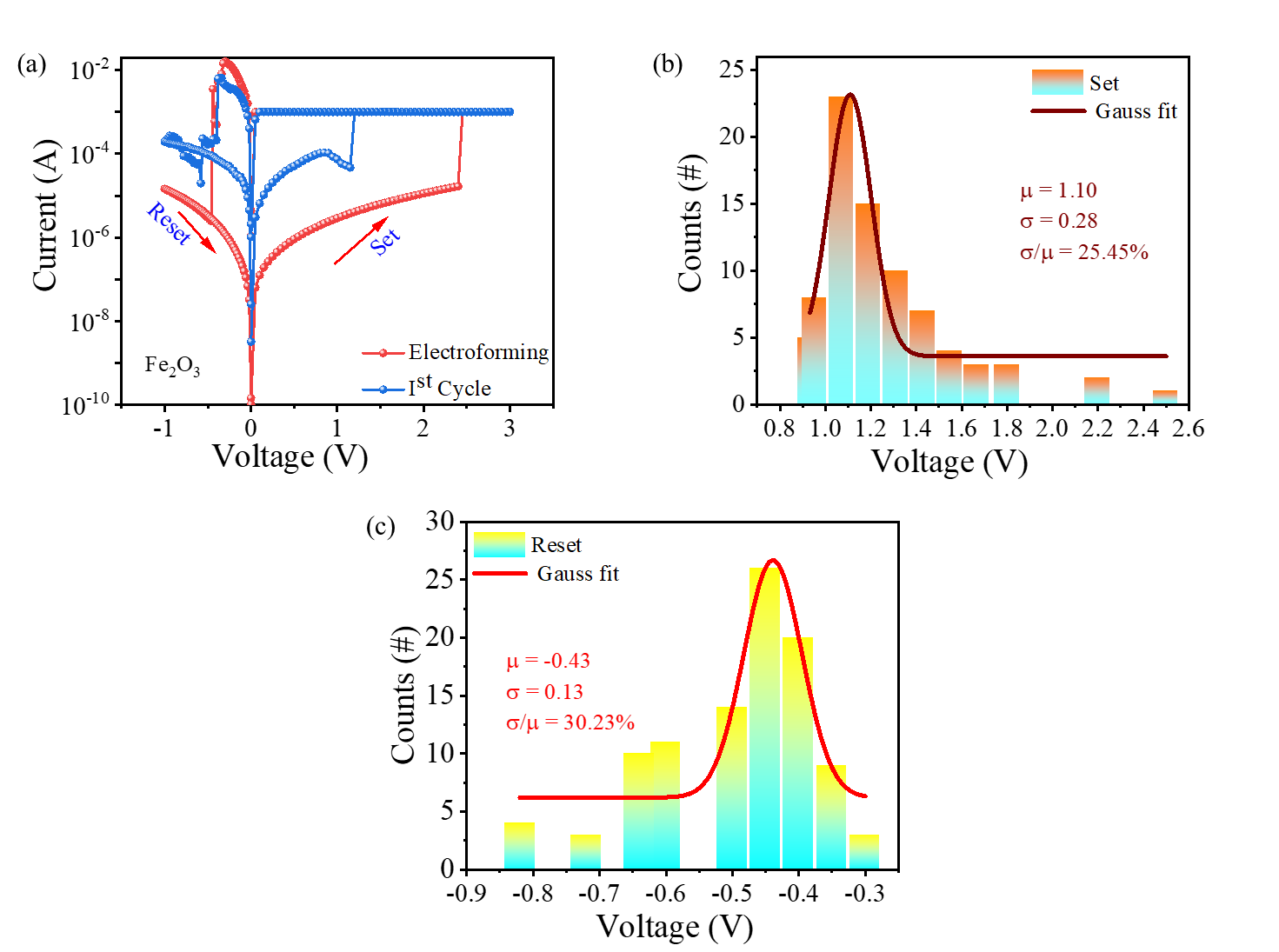
**

Figure S3: Electroforming and (a) I-V cycles of (Fe_2_O_3_) and histogram of (b) Set voltage and (c) reset volage.

**
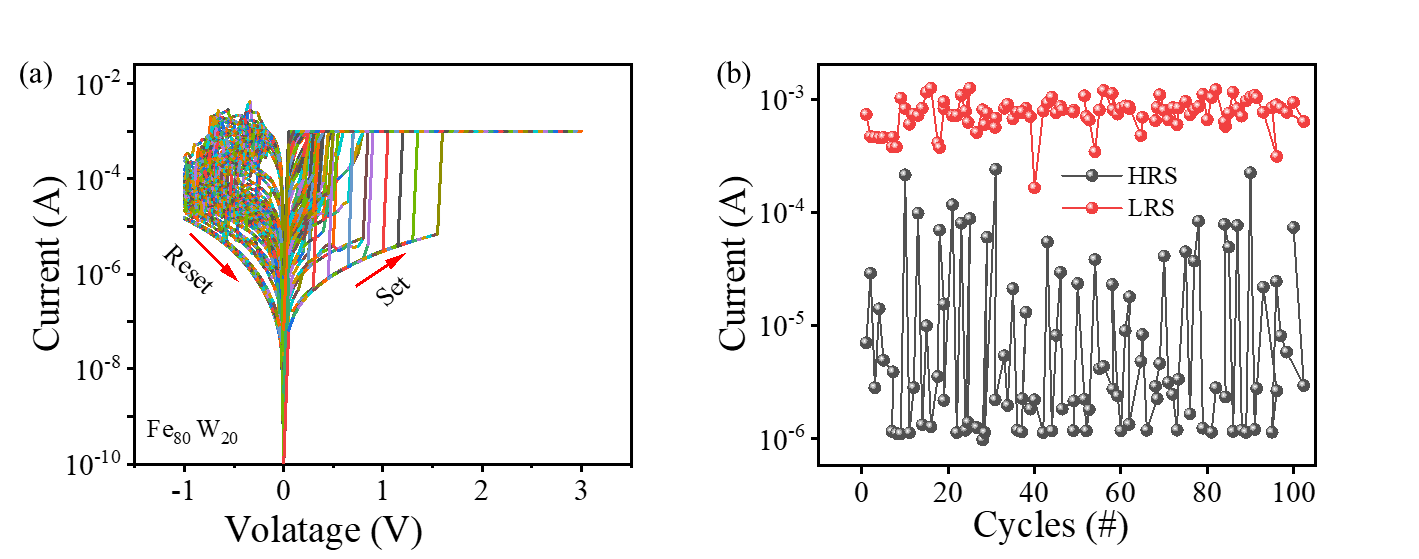
**

Figure S4(a-b): (a,b) Multiple I-V cycles and the endurance of the Ag/Fe_80_W_20_/Pt hybrid nanocomposites.

**
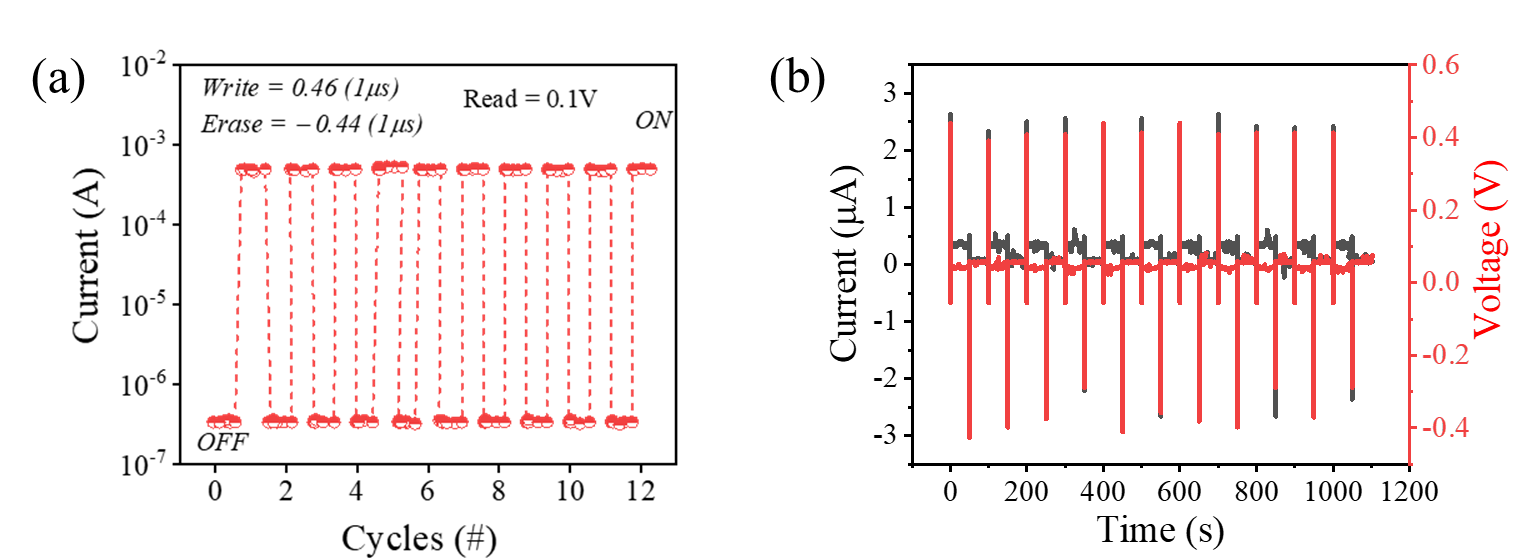
**

Figure S5: (a,b) Pulse scheme of consecutive endurance cycle and retention up to 1000s.


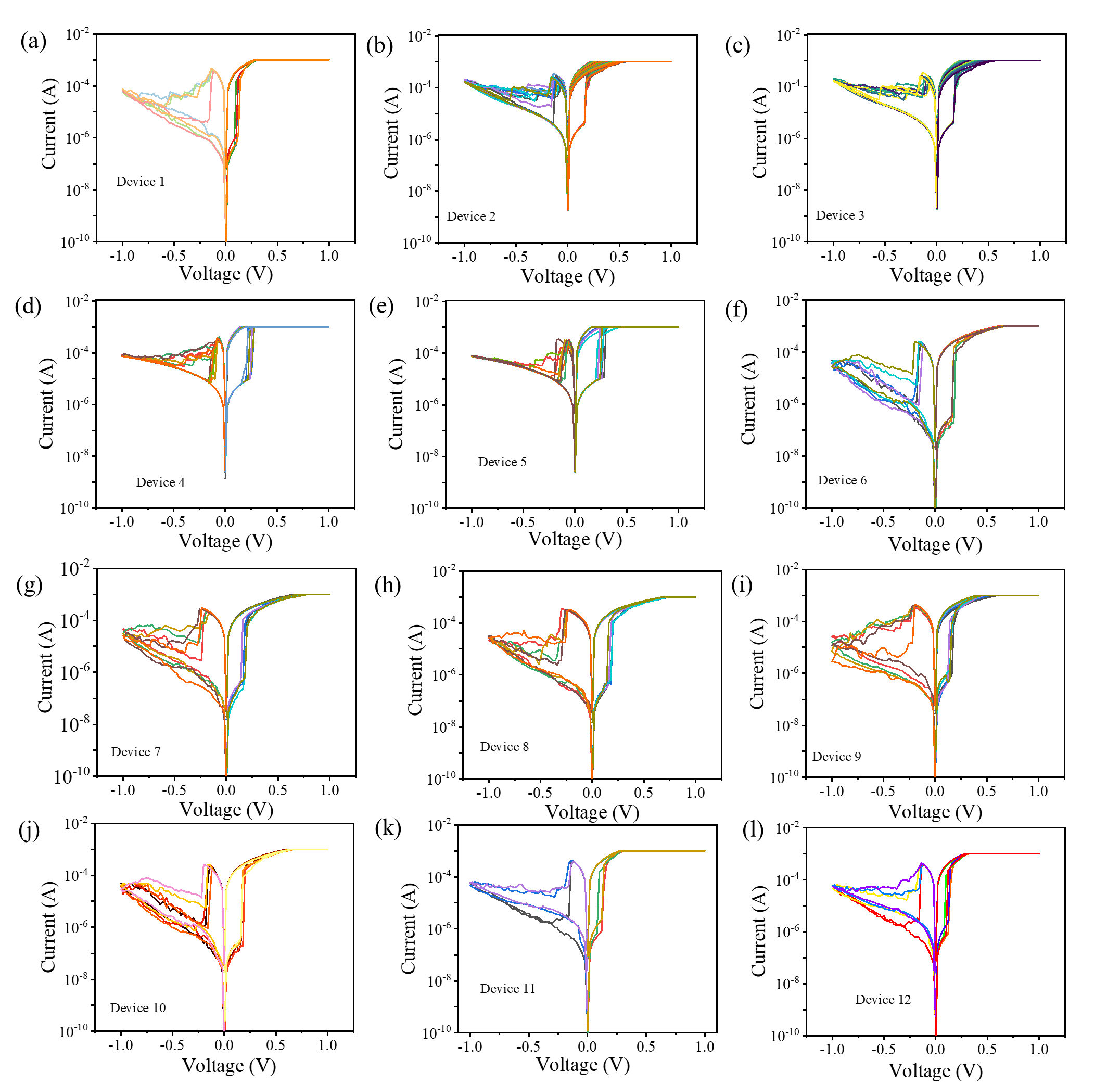


Figure S6: (a-l)To check the reproducibility and environmental stability of I-V cycles as-fabricate multiple devices.


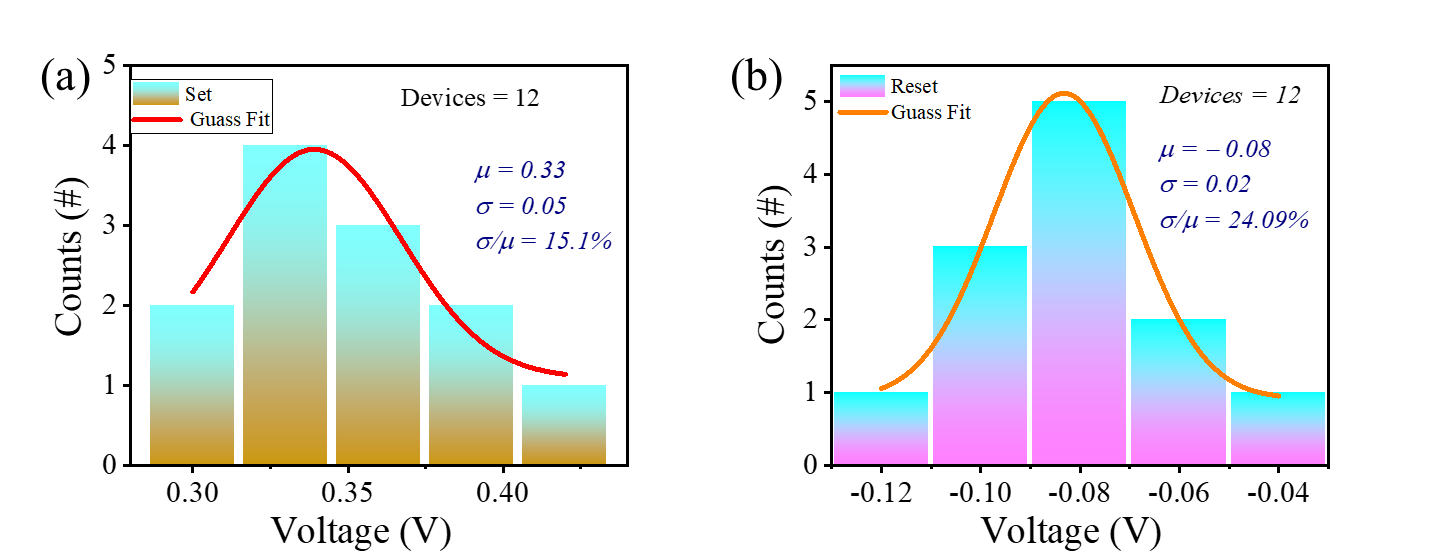


Figure S7: Device to device variations of Ag/Fe_90_W_10_/Pt hybrid nanocomposite of twelve fabricated devices (a) Set voltage (b) Reset voltage.

Figure S8: High resistance states (HRS) states of the twelve devices at different stopping voltages. .

Figure S9: The set and reset pulse to calculate the energy consumption of Ag/Fe_90_W_10_/Pt hybrid nanocomposite device.


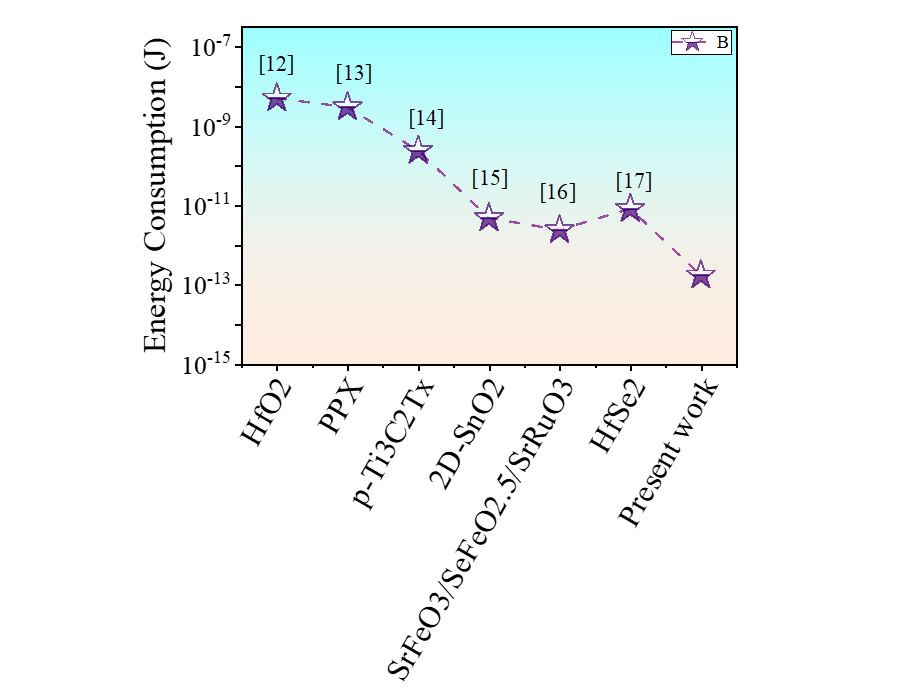


Figure S10: Performance metrics of the synaptic device compared with existing literature.

Figure S11: I-V cycles of Ag/Fe_90_W_10_/Pt device structure after six months to check the stability of device.

**
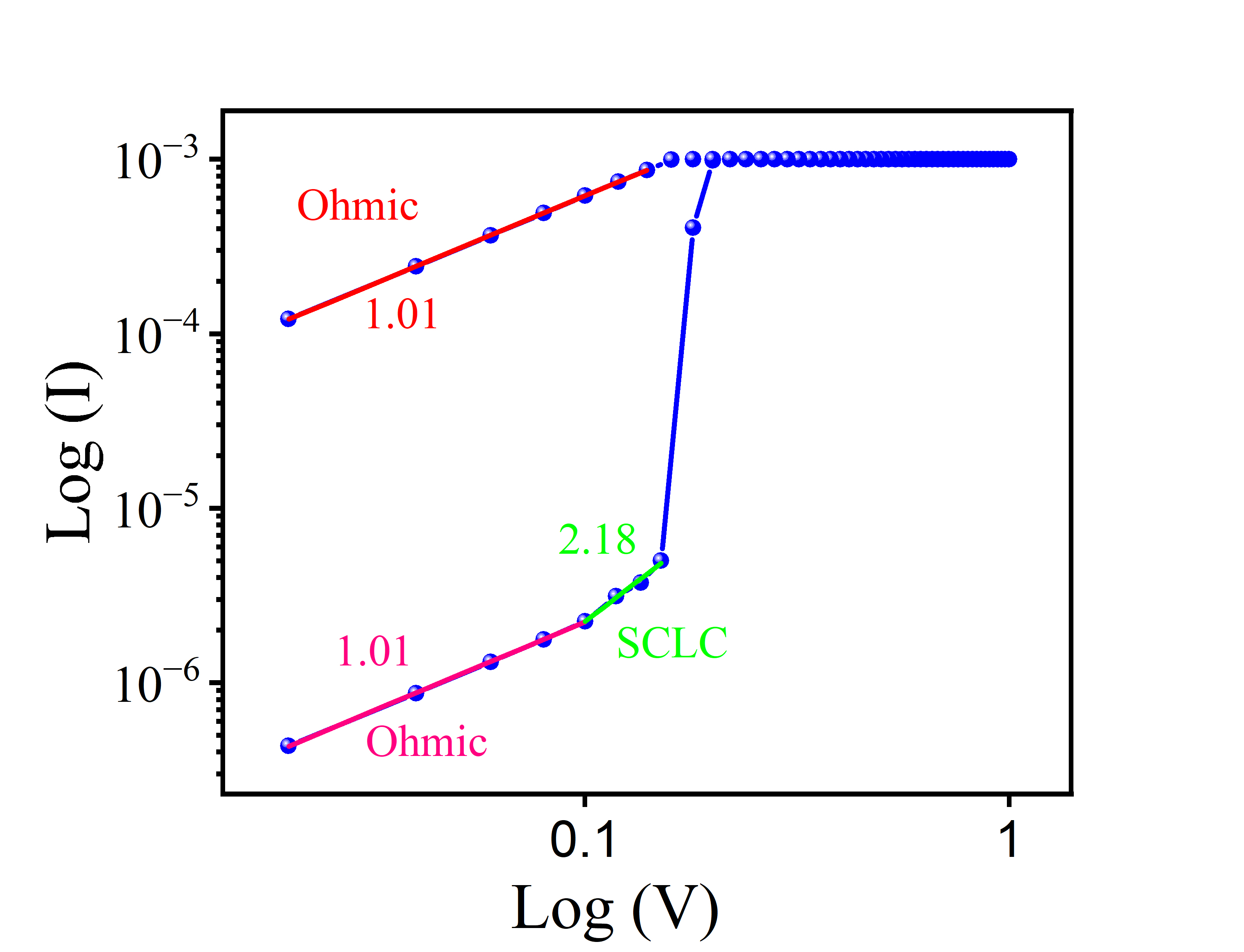
**

Figure S12: Conduction mechanism of the hybrid nanocomposite Fe_90_W_10_.


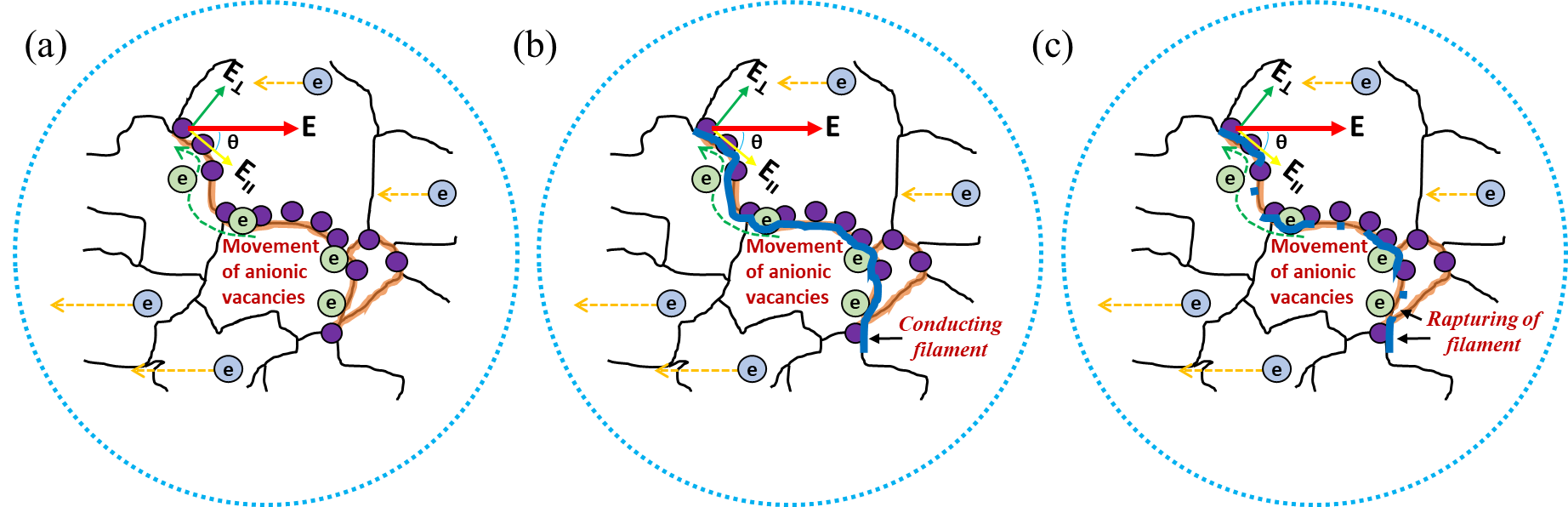


Figure S13: Schematic illustration of conducting and rapture of filament through grain boundaries.

Figure S14: Digital set vs-controlled analog reset I-V curves by varying the reset voltage from ‒0.3V to ‒ 0.7V having 0.1 increment size.


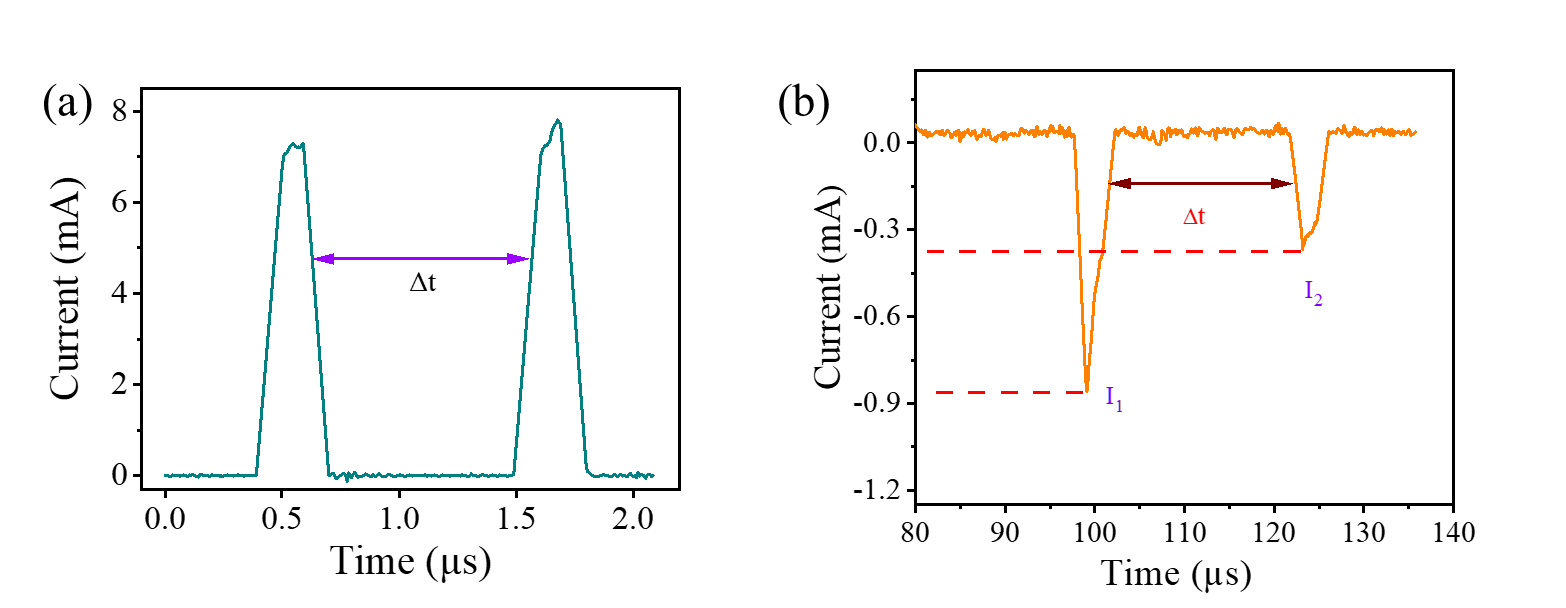


Figure S15: (a,b) Pulse scheme of (PPF) and paired-pulse depression (PPD).

**
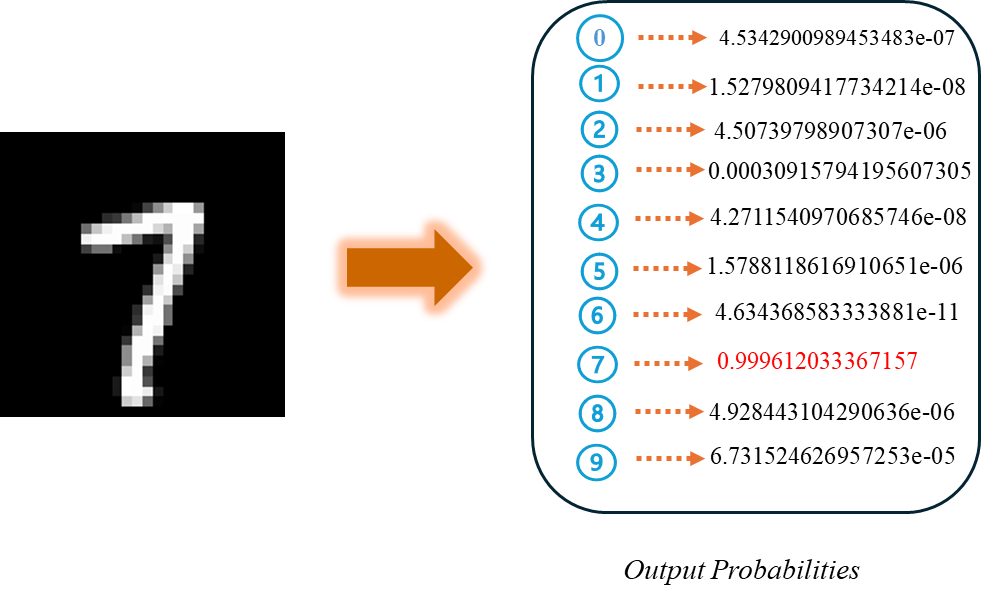
**

Figure S16: The recognition performance of the artificial neural network (ANN) was evaluated based on the probability assigned to each class.

Figure S17: MNIST digit set recognition accuracy with device-to-device effect variation.
